# Legal immigrants: invasion of alien microbial communities during winter occurring desert dust storms

**DOI:** 10.1186/s40168-017-0249-7

**Published:** 2017-03-10

**Authors:** Tobias Weil, Carlotta De Filippo, Davide Albanese, Claudio Donati, Massimo Pindo, Lorenzo Pavarini, Federico Carotenuto, Massimiliano Pasqui, Luisa Poto, Jacopo Gabrieli, Carlo Barbante, Birgit Sattler, Duccio Cavalieri, Franco Miglietta

**Affiliations:** 1 Research and Innovation Centre, Fondazione Edmund Mach, Via E. Mach 1, 38010 San Michele all’Adige, Trento Italy; 2 Institute of Biometeorology, National Research Council (IBIMET-CNR), Via Caproni 8, 50145 Florence, Italy; 3 Institute of Agricultural Biology and Biotechnology, National Research Council (IBBA-CNR), Via Moruzzi 1, 56124 Pisa, Italy; 4 Institute for the Dynamics of Environmental Processes, National Research Council (IDPA-CNR), Via Torino 155, 30172 Mestre, Venice Italy; 5 Department of Environmental Sciences, Informatics and Statistics, University Ca’ Foscari of Venice, Via Torino 155, 30172 Mestre, Venice Italy; 6 Institute of Ecology, University of Innsbruck, Technikerstraße 25, 6020 Innsbruck, Austria; 7 Department of Biology, University of Florence, Via Madonna del Piano 6, 50019 Sesto Fiorentino, Florence Italy

**Keywords:** Desert dust storm, Microbial ecology, Airborne pathogens, Long-distance dispersal, Global warming, Climate change, Ecosystem and public health, Metagenomics, Alpine soils, Invasion

## Abstract

**Background:**

A critical aspect regarding the global dispersion of pathogenic microorganisms is associated with atmospheric movement of soil particles. Especially, desert dust storms can transport alien microorganisms over continental scales and can deposit them in sensitive sink habitats. In winter 2014, the largest ever recorded Saharan dust event in Italy was efficiently deposited on the Dolomite Alps and was sealed between dust-free snow. This provided us the unique opportunity to overcome difficulties in separating dust associated from “domestic” microbes and thus, to determine with high precision microorganisms transported exclusively by desert dust.

**Results:**

Our metagenomic analysis revealed that sandstorms can move not only fractions but rather large parts of entire microbial communities far away from their area of origin and that this microbiota contains several of the most stress-resistant organisms on Earth, including highly destructive fungal and bacterial pathogens. In particular, we provide first evidence that winter-occurring dust depositions can favor a rapid microbial contamination of sensitive sink habitats after snowmelt.

**Conclusions:**

Airborne microbial depositions accompanying extreme meteorological events represent a realistic threat for ecosystem and public health. Therefore, monitoring the spread and persistence of storm-travelling alien microbes is a priority while considering future trajectories of climatic anomalies as well as anthropogenically driven changes in land use in the source regions.

**Electronic supplementary material:**

The online version of this article (doi:10.1186/s40168-017-0249-7) contains supplementary material, which is available to authorized users.

## Background

Many microorganisms use Aeolian dispersal as a key strategy for colonization of new habitats, and here especially, the global dispersal of microbial pathogens constitutes a threat to environmental health [1, 2]. Dust particles can facilitate intercontinental movement by acting as vehicles for viable microorganisms [3]. West African soils, primary the Saharan desert, are major sources of airborne dust that is frequently transported at high altitude over the Atlantic and the European continent [4]. Both the Sahara and the atmosphere are characterized by harsh conditions with limited availability of nutrients, high radiation, and extreme temperatures [5]. Therefore, microorganisms that are able to survive in these environments represent potential invaders of sites with comparable challenging conditions such as high-elevated alpine regions [6]. Further, recent knowledge suggests that the transported mineral and biological particles of large-scale dust storms affect cloud ice formation and precipitation processes, creating novel climate system feedbacks [7]. In particular, during winter, alien biological particles associated with large dust events may be deposited in vast numbers leaving persistent dust layers in the snowpack [8] that, at the highest elevations are preserved in the ice of glaciers. Such permanently frozen environments harbour a variety of viable microorganisms [9] that have been deposited over centuries [10], but it was rarely shown that they can persist in the new habitat after the snowpack was melted [11]. Previous studies revealed that microorganisms transported with Saharan dust can provoke several severe plant diseases [12]. Moreover, increasing incidences of pulmonary and cardiovascular diseases [13], as well as meningitis epidemics [14], have been attributed to dust storms either caused by the dust itself or due to plant-derived constituents or endotoxins of microbial dust “passengers” [1]. Hence, the characterization of alien microorganisms that are currently sequestered in the cryosphere is a priority while considering global warming and future trajectories of climatic anomalies as well as anthropogenically driven changes in land use in the source regions [4, 15, 16]. An increasing frequency of dust storms may intensify the global dispersal, the deposition, and consequently the rapid contamination of new habitats by alien microbes. Yet difficulties in separating dust associated from “domestic” microbes have complicated a precise description of the deposited airborne desert dust microbiota. To efficiently study the long-distance dispersal of microbes by large-scale desert dust events and their impact on distant ecosystems, a collaborating effort of geologists, atmospheric chemists, and microbiologists was strongly requested [3].

**Fig. 1:**
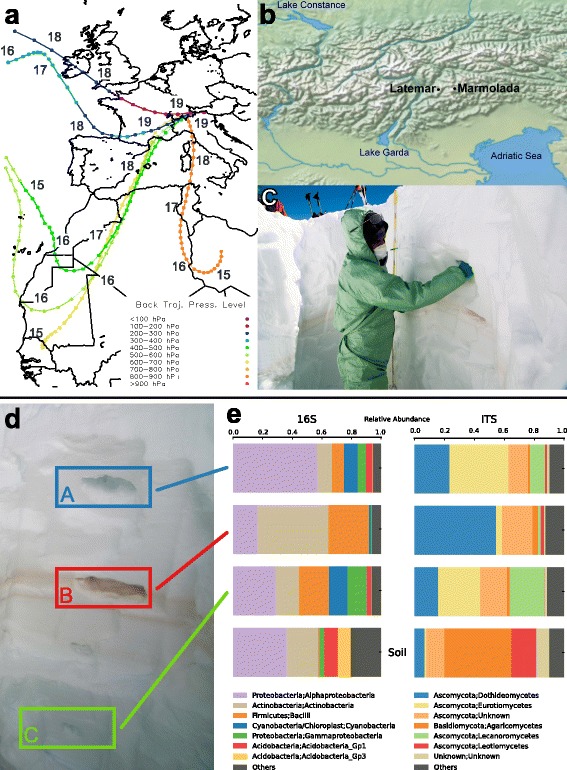
Saharan dust event in the Dolomite Alps on February 19th 2014. **a** Back trajectories computed at different height levels and expressed in pressure values (*color scale*: <100 and >900 hPa).The starting point is located at Long 11.81° and Lat 46.86°, on 19th of Feb 2014 12:00 UTC. Solid circles represent locations every 6 h and numbers refer to date (day of February). **b** *Map* (made with Natural Earth) showing collection sites in the Dolomite Alps. **c** Snow samples were collected wearing protective suits, facemasks, and vinyl gloves. **d** Snowpit (2 m depth) consisting of layer A: snow that fell after the Saharan dust event; layer B: Saharan sand-containing snow, clearly distinguishable by its light brown color. and layer C: snow that fell before the Saharan dust event. **e** Relative abundances of the most abundant bacterial (16S rRNA gene) and fungal (ITS) classes present in the three snow layers and the soil layer (not shown). Saharan sand-containing snow is characterized by a higher relative abundance of Actinobacteria and Dothideomycetes

In winter 2014, a massive amount of Saharan dust particles was mobilized and transported towards the Western Mediterranean Basin (Fig. 1) resulting in the largest Saharan winter dust event that was recorded at the Italian Climate Observatory [17] up to that time. This massive event was then efficiently deposited in the Alpine region, covered by successive snowfalls and thus remained sealed between two dust-free snow layers (Fig. 1), preventing windblown contamination by material from the local environment [18].The clear separation of snow layers associated with the dust event and with the dust-free snowfall events gave us the unique opportunity to determine with high precision the bacterial and fungal communities that were transported in the troposphere from the Sahara to the Alps (Fig. 1). Additionally, we tested the contamination potential of these airborne communities by comparing them to the Alpine soil microbiota after snowmelt.

Our detailed metagenomic analysis unveiled that extreme meteorological events can move not only fractions but also large parts of entire microbial communities over continental scales, that this microbiota contains highly destructive environmental and important human pathogens, and that winter occurring dust depositions bear a great potential for a rapid microbial contamination of sensitive sink habitats after snowmelt.

## Results

### Back trajectories

During the wetter than normal winter 2014 over the Alpine region by the end of January, a positive snow anomaly was present in the central sector 1500–1700 masl [19]. In this area, between the end of January and the first half of February abundant precipitation events provided a thick snow coverage even at low altitudes starting from 800 masl. [19]. On 14th of February, a cyclonic depression generated over the southern Morocco moved rapidly eastward to Mauritania, north Mali and south Algeria, mobilizing a massive amount of Saharan dust particles until 17th of February. Then, on 19–20 February 2014, a north–northwest displacement of the sub-tropical Azores high-pressure system over the Atlantic Ocean, and a large anticyclone circulation centered over Libya provided the favorable conditions for a north-to-south elongated trough, associated with an extratropical cyclone over the British Islands, that was extended from Senegal/Niger to Great Britain and Western Europe. It generated a vigorous southerly flow towards the Western Mediterranean Basin from February 14th to 20th (Fig. 1, Additional file 1: Figure S1) resulting in the largest Saharan winter dust event ever recorded at the Italian Climate Observatory [17]. This massive aerosol plume was then efficiently deposited during a moderate snowing event at altitudes above 1800 masl. in the Alpine region on 19th of February. The Saharan dust-rich snow layer was covered by successive snowfalls, starting 2 days later due to Atlantic air masses and thus remained sealed between two dust-free snow layers (Fig. 1). Backward trajectories, based on a high-resolution non-hydrostatic mesoscale model simulation [19], revealed that the most likely emission source was located along the path covering Mali, Mauritania, Morocco, and Algeria (Fig. 1).

### Geochemistry

The major ion composition of deposited mineral dust validated the back trajectories (Fig. 1) for the identification of the potential source areas (Table 1, Additional file 1: Table S1). Geochemical characterizations clearly showed that the chemical composition of the snow containing Saharan dust differed from dust-free snow for higher concentrations of trace, major, and rare earth elements, in particular regarding the very high concentrations of crustal elements such as Na, Mg, K, Ca, and Fe (Additional file 1: Figure S2). Elemental ratios are one of the best tools to reveal regional potential source areas of Saharan dust particles [20]. In this case, the elemental mass ratios of snow samples containing dust (MA289 and MAJ025) are within the intervals reported for the Saharan desert [21] (Table 1).

**Table 1:** Comparison of crustal element ratios. Comparison between values of crustal element ratios measured in bulk sediments from North Africa (Sahara desert, Scheuvens et al. [20]) and the same ratios measured in Marmolada samples (MA289 and MAJ025)

	Sahara desert	Sample MA289	Sample MAJ025
*Ca/Al*	2.36–6.06	3.97	4.31
*Fe/Al*	0.60–0,88	0.67	0.77
*Mg/Al*	0.34–1.54	1.17	1.10
*Na/Al*	0.07–0.44	0.70	1.07
*K/Al*	0.20–0.33	0.70	0.70
*(Ca + Mg)/Fe*	4.29–8.40	8.10	6.70

Detailed chemical compositions of snow samples are given in Table S1

### Metagenomics

The pyrosequencing produced a total of 310.994 reads of the V3-V5 variable region of the 16S rRNA gene (average read length 649 nt) and 582.811 reads of the variable ITS1 region (average read length 443 nt). After pre-processing and rarefaction, a total of 1282 operational taxonomic units (OTUs) for the 16S rRNA gene (with a depth of 2879 reads per sample) and 1095 OTUs for ITS data (with a depth of 5264 reads per sample) were considered.

**Fig. 2:**
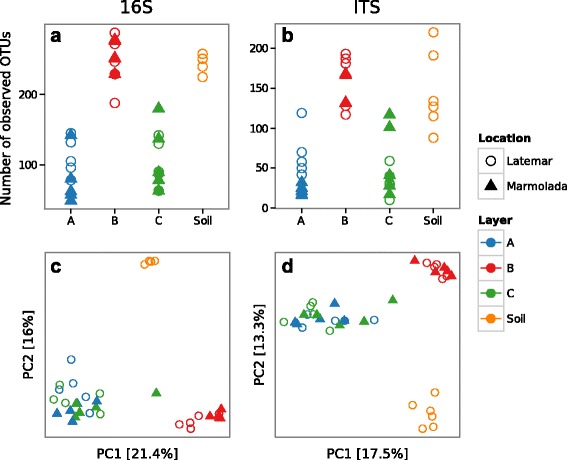
Community diversity between the three snow layers and soil samples. The measures of α-diversity for each sample are given. **a** Number of observed bacterial 16S rRNA gene OTUs. **b** Number of observed fungal ITS1 OTUs. PCoA of the between samples distances, for **c** bacterial and **d** fungal samples, were measured using unweighted UniFrac distance. Symbol *shapes* indicate the two Dolomite mountain collection sides. *Colors* of snow layers are coded according to Fig. 1d and soil samples are given in *orange color*

Both the targeted 16S rRNA gene and ITS1 sequencing revealed clear differences between the bacterial and fungal communities present in snow containing Saharan sand (layer B) and snow that fell before (layer C) and after (layer A) the deposition event (Fig. 1; Fig. 2, unweighted UniFrac distance PERMANOVA *P* < 0.0001). Layer B showed a significantly higher diversity of its microbial community than layers A and C (Fig. 2, *P* = 5.5 × 10^−4^ and *P* = 7.3 × 10^−4^ for the 16S rRNA gene and ITS, respectively, Wilcoxon rank-sum test, FDR corrected; Additional file 1: Figure S3). Layers A and C were characterized by a higher fraction of the bacterial classes Alpha-, Gammaproteo-, and Cyanobacteria (Fig. 1), with *Pseudomonas* as the most abundant genus (Additional file 1: Figure S4). We could additionally identify with high precision other microorganisms significantly enriched in dust-free snow, these are *Acinetobacter*, *Caulobacter*, *Acquabacterium*, *Beijerinckia*, *Novosphingobium*, *Granullicella*, *Terriglobus*, *Pelomonas*, *Ralstonia*, and *Phenylobacterium* (Additional file 1: Figure S4). Regarding fungi, layers A and C were especially rich in molds of the genus *Penicillium* (Additional file 1: Figure S5).

**Fig. 3:**
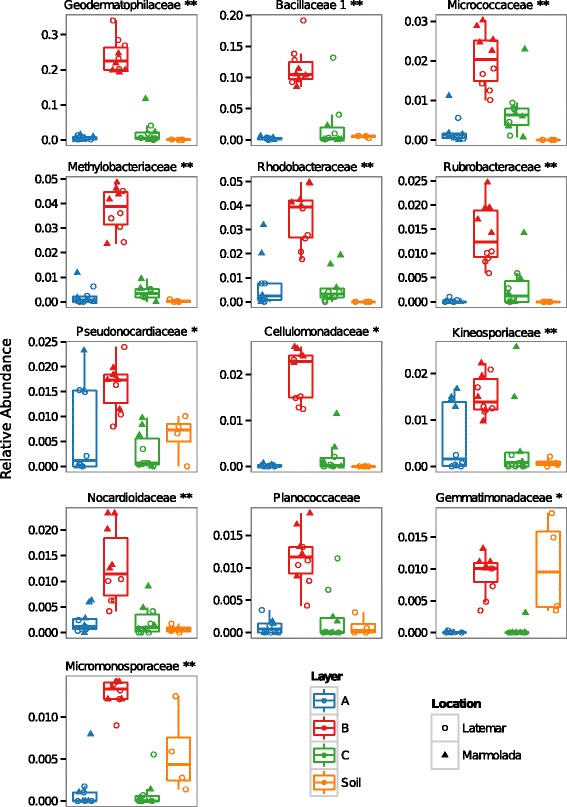
Significant enriched bacterial families (Wilcoxon rank-sum test, *P* < 0.05, FDR corrected) in Saharan sand-containing snow. Comparison of the sand-free snow layers (**A**) and (**C**) against the Saharan sand-containing snow layer (**B)**. *Asterisks* indicate presence among the most** and general* abundant families described for Saharan sand collected in Chad [22]. Families taken in consideration are present >1% in at least 20% of all samples

Snow containing Saharan dust (layer B) was mainly represented by Actinobacteria and Bacilli (Fig. 1). Strikingly, layer B was significantly enriched in the most abundant bacterial families previously described for Saharan sand samples taken in Chad [22] (Fig. 3), and we found that 85% of the principal Saharan bacterial genera [22] (Additional file 1: Figure S4) arrived in the European Alps in a single meteorological event. The genus *Geodermatophilus* was frequent in all layer B samples*.*

**Fig. 4:**
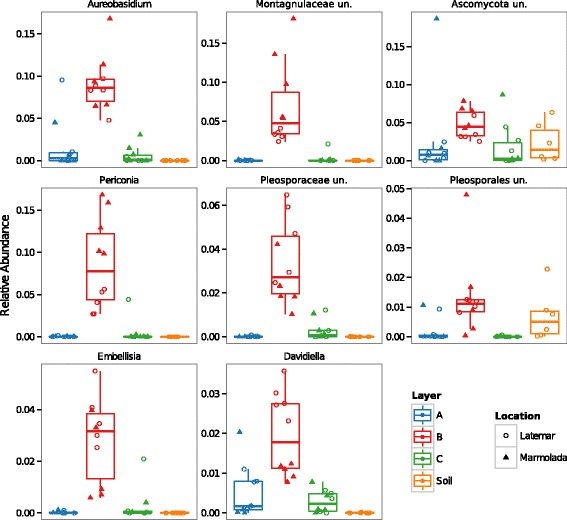
Significant enriched fungal genera (Wilcoxon rank-sum test, *P* < 0.05, FDR corrected) in Saharan sand-containing snow. Comparison of the sand-free snow layers **A** and **C** against the Saharan sand-containing snow layer **B**. *un* unidentified. Genera taken in consideration are present >1% in at least 20% of all samples

As seen for the long-range transport of bacterial communities, also the three most abundant fungal classes described for the Saharan desert [22], namely, Dothideomycetes, Agaricomycetes, and Sordariomycetes, were significantly enriched in layer B when compared to sand-free snow layers (FDR adjusted Wilcoxon rank-sum test *P* = 7.7.0 × 10^−4^, 2.5 × 10^−3^, and 2.0 × 10^−3^). Additionally, layer B snow could be easily distinguished from sand-free snow, as 79% of the significantly enriched fungal genera were almost exclusively present in layer B samples (Additional file 1: Figure S5). Like the mycobiota described for hot deserts, fungal microbes specific for dusty snow were dominated by imperfect ascomycetes, teleomorph genera, and dematiaceous fungi with darkly pigmented, thick-walled, and strongly melanized spores [23]. A closer look on these fungal genera unveiled that mainly so-called “black fungi” especially of the orders Pleosporales, Capnodiales, and Dothideales were dominant in almost all layer B samples as reflected in the genera, *Aurebasidium*, *Periconia*, *Pleosporaceae*, *Montagnulaceae*, *Embellisia*, and *Davidiella* (Fig. 4).

To uncover if alien dust storm microbes remained in the Alpine soil after snowmelt, we compared operational taxonomic units (OTUs) of species exclusively present in layer B with those present in soil samples. Out of these identical OTUs, 82 bacterial and 36 fungal OTUs matched the criterion (Additional file 2: Table S2). This is equivalent to approximately 10% of the bacterial and fungal layer B OTUs. Members of the fungal class Dothideomycetes seem to be predesignated for the survival in new challenging environments as half of the fungal species belonged to them with *Pleosporales* as the dominant order and *Phoma* as the most abundant genus (Additional file 2: Table S2). Regarding bacteria, Actinobacteria was the prevalent class (39%), followed by Alphaproteobacteria (22%), and Bacilli (9%) (Additional file 2: Table S2).

## Discussion

During last decades, an overall increase in quantity of African dust depositon was noted [24]. This phenomenon has been so far considered relevant for nutrient deposition and soil development [25, 26], yet the recent knowledge on the microbial world suggests that dust deposition may introduce exotic microorganisms, including pathogens, into sensitive ecosystems [3, 4]. First insights on the immigration potential of Saharan dust microbes has been gained from studies on high alpine remote lakes, highlighting microorganisms that might have the capability to be successful colonizers under favourable conditions [4, 27–29]. Here, we analyzed the microbial load of the largest ever recorded winter-occurring Saharan dust event in Italy and, for the first time, we provide evidence on the potential contamination of alpine soils by desert dust microorganisms after snowmelt.

The clear demarcation of the deposited dust allowed us to precisely determine microorganisms associated with this massive dust event. The role of desert storm sand deposition in moving entire microbial communities is mirrored by the result that layer B (dust-containing snow) has a significantly higher alpha diversity than layers A and C (dust-free snow). The African origin of these microbial communities is reflected in the high conformity of principal bacterial and fungal genera described for the Saharan desert [22] with those present in layer B. It is especially noteworthy that extremely stress-resistant bacteria and fungi were among the most abundant microbes present in the snow containing Saharan sand. They are known for their ability to adapt to environmental stressors including desiccation and extreme γ radiation, like in the case of the genus *Geodermatophilus* [30]. Several members of these black and thick-walled bacteria were isolated from arid soils [31], from Mont Blanc snow containing Saharan sand [6], and directly from sand sampled in the Saharan desert [22].

Further, endospore-forming *Bacillus* species are highly present in Saharan sand [22], and this genus was also frequently detected in our layer B samples. Similarly, viable *Bacillus* species originating from Chinese deserts were shown to be distributed via the atmosphere to long distance habitats in Japan [32, 33]. The ability to form spores provides resistance to extreme environments [34] and indicates that these genera have a high probability of surviving years of enclosure in the snowpack [35], while for the survival in the troposphere pigmentation seems to be an important factor [4, 8]. Regarding the latter case, the pigmented Deinococcus-Thermus and Gemmatimonadetes were recently considered as bio-indicators for Saharan dust events [8] which is supported by our observations (Additional file 1: Table S4). The genera *Deinococcus* and *Gemmatimonas* were significantly enriched in layer B, but Gemmatimonas was also enriched in soil samples. Gemmatimonadetes are normally not present in the Southern European air, but are prevailing bacteria in soils of recently deglaciated glacier forefields [8, 36], therefore they might represent a suitable example for Saharan immigrants of alpine soils.

The so-called black fungi were characteristic for the fugal dust-borne community. They are among the most stress-resistant organisms on Earth and colonize rock surfaces even in the most arid zones of the world [23]. The remarkable survival capacity of black fungi has been further highlighted by a recent study in which Saharan dust samples collected by Charles Darwin in 1838 were successfully cultured and here, next to spore-forming bacteria (e.g., *Bacillus*), a member of Dothideomycetes (*Davidiella tassiana*, Capnodiales) was isolated among cultivable fungi [37]. More important Capnodiales and Pleosporales contain several highly destructive and important pathogens of cereal crops, vegetables fruits, dicots, and trees as well as the most important fungal allergens [38].

Taken together, Saharan sand microbes that arrived with the dust event showed the potential to overcome all challenges posed, such as long-distance transport in the troposphere, long quiescence state in snow at sub-zero temperature, low nutrient availability, and persistence in the Alpine soil after snowmelt. Representatives of black thick-walled fungi and bacteria seem to have the best armamentarium, which allows them to survive harsh conditions. This high adaptation potential to challenging environments in a changing climate might be a possible prerequisite for the evolution of pathogenicity [23]. Moreover, pathogens cannot solely survive in foreign environments but some can still germinate as it is the case for some ancient viruses recovered from melted permafrost in which it has been stored for more than 30,000 years [39]. The “European Respiratory Society Environment and Health Committee” recommended attention and control of wind-blown dust from, for example, the Sahara, as they may have adverse health effects [40]. Hence, together with fine inorganic dust particles, dust-associated microbes represent a serious health risk as several layer B genera include species that are known to cause allergic reactions, pulmonary infections, skin infections, or fatal brain infections in humans as well as leaf disease, inhibition of growth, branch and stem canker, blight, rot, scab, and mildew in plants. On the other hand, it has to be noted that some of these microbes are, albeit their potential pathogenicity, known as beneficial organisms like members of the genus *Aurebasidium* [41, 42].

The dust-free snow (layers A and C) was significantly enriched on genera containing known ice nucleators such as *Pseudomonas*, that were missing from layer B. The high relative abundance of *Pseudomonas* in dust-free snow samples might be related to the ability of some *Pseudomonas* species to initiate ice formation by special ice nucleation active proteins present in their outer membrane [43]. Next to *Pseudomonas*, dust-free snow was significantly enriched in *Acinetobacter* and molds of the genera *Penicillium. Acinetobacter* genera were frequently found in atmospheric water, and *Penicillium* strains were discussed to impact the atmospheric multiphase chemistry [44]. Additionally, we could identify other microorganisms significantly enriched in dust-free snow, and it will be interesting to study whether they have ice nucleation activities similar to *Pseudomonas* or be hitchhiking on ice nucleators. Our data supports the hypothesis that Saharan dust and biological aerosols have ice nucleation activity and are important for precipitation processes [45]; it further suggests that dust-driven snowfalls seem to be events that are microbiologically much more complex and well separated from dust-free snowfalls, bearing the potential ability of the former to bring down to the ground species that normally are not airborne, at least on such big distances.

## Conclusions

Overall, there is evidence that large dust storms can move *quasi*-entire microbial communities far away from their area of origin and thus subsequently exposing them to novel environmental niches. The inoculum size is crucial as it determines the ability of a microbial community to survive, colonize, and eventually take over in a new environment. When dust events occur during the warm seasons, in form of rain, these are likely buffered by the robustness of the recipient microbial communities, and the inoculum is likely to be diluted and dispersed by surface runoff and infiltration. But when these events occur during winter, the microbes are accumulated in the snow, reaching a high titer upon rise of temperature during spring and summer. In those cases, masses of microbes are released in high concentrations on a limited surface during the melting of the snowpack. This effect might be further multiplied by the release of similar alien microbes that have been transported with desert dust over centuries and that were stored in the now ever retrieving glacial ice [9]. The quantification of the overall load of microorganisms currently sequestered in melting glaciers should be assessed by means of ice cores sampling. Awareness of this realistic microbial contamination together with the increasing availability of next generation sequencing resources for metagenomic analysis, require the development of up-to-date monitoring capabilities targeted to melting snow and glacier effluents for the effective implementation of early warning systems.

## Methods

### Back trajectories

A full non-hydrostatic regional model (Regional Atmospheric Modeling System (RAMS)) has been forced by atmospheric fields from NCEP/NCAR Reanalysis dataset and sea surface temperature field obtained from the Optimal Interpolation Sea Surface Temperature dataset. The model simulation was run over a wide domain covering North Atlantic basin, Africa, Europe, and Middle East, from February 1s to March 1st 2014, with a horizontal grid spacing of 50 km, a vertical extent of about 21 km above sea level, and a temporal resolution of 1 h. Backward trajectories calculation has been computed by using the full 3D wind field modelled by the RAMS and with a centred difference in time computation in Grid Analysis and Display System (GrADS) (http://cola.gmu.edu/grads/grads.php). This regional reanalysis approach guarantees a coherent description of the three-dimensional flow dynamics. Furthermore, the non-hydrostatic formulation of model, along with the fine time resolution (1 h) ensures a reliable representation of backward trajectories [46].

### Sampling sites

Samples were collected on two mountains located in the Trentino region of the Italian Alps: Marmolada (Punta Rocca, collection site at 3054 m) and Latemar (collection site at 2080 m) (Fig. 1). To maximize sampling efficiency, we collected at an altitude for which the maximum deposition of Saharan dust (approx. 2000–3000 m) was reported [47]. Further, the air temperature at the collection sides rarely, if at all (Marmolada), exceeds freezing during winter. Snow samples were collected based on established sampling methods [18, 48]. Briefly, samples were taken wearing protective suits (coveralls), facemasks, and vinyl gloves. Surface snow was removed using a clean sterilized shovel. The snowpit was carefully dug in order to maintain the stratigraphy of the snow layers and consisted of layer A: snow that fell after the Saharan dust event; layer B: Saharan sand-containing snow, clearly distinguishable by its light brown color, and layer C: snow that fell before the Saharan dust event (Fig. 1).The compacted snow of the layers indicated that no snowmelt took place after dust deposition. Samples were collected in sterile plastic containers or plastic bags, were transported on dry ice, and were stored at−80 °C.

### Geochemistry

Two snow samples with clearly visible dust layers and four sample of “clean” snow were collected at different depths from the entire pit profile at the Marmolada collection site. In the laboratory, samples were melted under a clean bench and acidified with HNO_3_ (ultrapure grade, Romil, Cambridge, UK) to obtain 2% solutions (v/v). CRC-ICP-MS measurements were performed using an Agilent 7500cx collision/reaction cell inductively coupled plasma mass spectrometer (CRC-ICP-MS) equipped with a CETAC ASX-520 auto-sampler. Measurements of selected major and trace elements were carried out with and without the collision cell in both helium and in hydrogen mode to reduce potential interferences. Instrumental drift and plasma fluctuations were corrected by online addition of a Rhodium internal standard solution (0.1 mg L^−1^, Ultra Scientific, Milano, Italy, 1000 mg L^−1^ stock solution).

The elemental suite was quantified using an external calibration. Five calibration standards ranging from 0.1 to 100 μg L^−1^ were prepared from the 10 mg L^−1^ multi-elemental standards CLM-2AN and IMS-101 (Ultra Scientific, Milano, Italy). Three additional calibrations points were prepared for the major crustal elements (Fe, Al, Na, K, Ca, Mg, Ti) from single standard solutions (ULTRA Scientific, 1000 mg L^−1^). The concentrations of these ranged from 200 to 2000 μg L^−1^.

For quality control purposes a certified reference material (CRM TMRAIN-04, lot 0913, Envir. Canada) was analyzed. The measured values are in good agreement with the certified and information values for this CRM (Additional file 1: Table S3).

### Metagenomics

Per collection site, five snow samples were taken. A snow sample set consists of three samples: layer A—fresh snow above the Saharan sand; layer B—snow containing Saharan sand; and layer C—snow below the Saharan sand layer (Fig. 1). Depending on snow height on the two mountains, layer A was collected from 30 to 60 cm, layer B from 70 to 100 cm, and layer C from 120 to 180 m under the snow surface. Additional six soil samples were taken at the Latemar collection site after snowmelt in a depth of approximately 10 to 20 cm under the surface.

All samples were gently melted and opened inside a clean bench. Melted snow (approx. 30 ml) was filtered (MO BIO Water filter; 0,22 μm), and genomic DNA was extracted using the PowerWater® DNA Isolation Kit (MO BIO Laboratories Inc., Carlsbad, USA) according to the manufacturer’s instructions. Soil samples were extracted using the FastDNA Spin Kit for Soil (MP Biomedicals, Santa Ana, USA) according to the manufacturer’s instructions. DNA quality was assessed by gel electrophoresis and UV-Vis spectroscopy. High-throughput metagenomics sequencing of the variable V3-V5 region of the bacterial 16S rRNA gene and of the fungal ITS1 region was performed on a GS FLX+ system using the XL+ chemistry following the manufacture’s protocols.

Raw 454 files were demultiplexed using the Roche’s sff file software. Reads were pre-processed using the MICCA pipeline [49]. Operational taxonomic units (OTUs) were assigned by clustering the sequences with a threshold of 97% pair-wise identity, and their representative sequences were classified using the RDP [50] software version 2.8.

Alpha (within-sample richness) and beta diversity (between-sample dissimilarity) estimates were computed using the phyloseq R package [51]. Permutational MANOVA (PERMANOVA) statistical tests were performed using the R package vegan [52] (adonis function) with 9999 permutations. See supplementary information for full detailed methods.

## Additional file

Additional file 1: Supplementary Information [53–56]. (PDF 7920 kb)
Additional file 2: Supplementary Information - Table S2. (XLSX 30 kb)
Additional file 3: Supplementary Information - Table S5. (XLSX 16 kb)
