# Supplementary material for: Legal immigrants: invasion of alien microbial communities during winter occurring desert dust storms
Source: Microbiome. 2017 Mar 10;5:32. doi: 10.1186/s40168-017-0249-7 (PMC5345179; doi:10.1186/s40168-017-0249-7)
Supplement: Additional file 1: — Supplementary Information [53–56]. (PDF 7920 kb) [file 40168_2017_249_MOESM1_ESM.pdf]

Supplementary Information

Legal immigrants: Invasion of alien microbial communities during winter occurring desert dust storms.

Tobias Weil, Carlotta De Filippo, Davide Albanese, Claudio Donati, Massimo Pindo, Lorenzo Pavarini, Federico Carotenuto, Massimiliano Pasqui, Luisa Poto, Jacopo Gabrieli, Carlo Barbante, Birgit Sattler, Duccio Cavalieri, Franco Miglietta.

correspondence to: duccio.cavalieri@unifi.it or f.miglietta@ibimet.cnr.it

APPENDICES:

Full Supplementary Methods

Additional file 1: Figures S1 – S5

Additional file 1: Tables S1 – S4

Additional file 1: References

Full Supplementary Methods

Back trajectories

A full non-hydrostatic regional model (Regional Atmospheric Modeling System (RAMS)) has been forced by atmospheric fields from NCEP/NCAR Reanalysis dataset and sea surface temperature filed obtained from the Optimal Interpolation Sea Surface Temperature dataset. The model simulation was run over a wide domain covering North Atlantic basin, Africa, Europe and Middle East, from February 1st to March 1st 2014, with a horizontal grid spacing of 50km, a vertical extent of about 21km above sea level and a temporal resolution of 1 h. Backward trajectories calculation has been computed by using the full 3D wind field modelled by the RAMS and with a centred difference in time computation in Grid Analysis and Display System (GrADS) (http://cola.gmu.edu/grads/grads.php). This regional reanalysis approach guarantees a coherent description of the three dimensional flow dynamics. Furthermore the non-hydrostatic formulation of model, along with the fine time resolution (1 h) ensures a reliable representation of backward trajectories [46].

Sampling sites

Samples were collected on two mountains located in the Trentino region of the Italian Alps: Marmolada (Punta Rocca, collection site at 3054m) and Latemar (collection site at 2080m) (Fig.1). To maximize sampling efficiency, we collected at an altitude for which the maximum deposition of Saharan dust (approx. 2000-3000m) was reported [47].

Geochemistry

Two snow samples with clearly visible dust layers and four sample of "clean" snow were collected at different depths from the entire pit profile: 30cm (MAJ037), 60cm (MAJ062), 80cm (MA375), 85cm (MA289), 93cm (MAJ025) and 120cm (MA131). Presence of dust particles was clearly visible for samples MA289 and MAJ025. All sampling tools and low-density polyethylene (LDPE) bottles were pre-cleaned with ultra-pure HNO_3 (Ultrapure grade, Romil, Cambridge, UK) and then rinsed several times with ultra-pure water. Snow was sampled into LDPE vials which were immediately capped, packed in double LDPE bags and transported to laboratory where samples remained frozen until the analysis. In the laboratory samples were melted at room temperature in LDPE sampling vials in a class 100 laminar flow clean bench and approximately 10 mL aliquots of each samples were transferred to 15 mL ultra-clean LDPE vials and acidified with HNO_3 (Ultrapure grade, Romil, Cambridge, UK) to obtain 2% solutions (v/v). CRC-ICP-MS measurements were performed using an Agilent 7500cx collision/reaction cell inductively coupled plasma mass spectrometer (CRC-ICP-MS) equipped with a CETAC ASX-520 auto-sampler. Measurements of selected major and trace elements were carried out with and without the collision cell in both helium and in hydrogen mode to reduce potential interferences. Instrumental drift and plasma fluctuations were corrected by online addition of a Rhodium internal standard solution (0.1 mg L^{-1}, Ultra Scientific, Milano, Italy, 1000 mg L^{-1} stock solution).

The elemental suite was quantified using an external calibration. Five calibration standards ranging from 0.1 to 100 µg L^{-1} were prepared from the 10 mg L^{-1} multi-elemental standards CLM-2AN and IMS-101 (Ultra Scientific, Milano, Italy), containing respectively Ag, Al, As, Ba, Be, Ca, Cd, Co, Cr, Cs, Cu, Fe, Ga, K, Li, Mg, Mn, Na, Ni, Pb, Rb, Se, Sr, Tl, U, V, Zn and Ce, Dy, Er, Eu, Gd, Ho, La, Lu, Nd, Pr, Sm, Sc, Tb, Th, Yb, Y. Three additional calibrations points were prepared for the

major crustal elements (Fe, Al, Na, K, Ca, Mg, Ti) from single standard solutions (ULTRA Scientific, 1000 mg L^{-1}). The concentrations of these ranged from 200 to 2000 μg L^{-1}.
For quality control purposes a certified reference material (CRM TMRAIN-04, lot 0913, Envir. Canada) was analyzed. The measured values are in good agreement with the certified and information values for this CRM (Additional file 1: Table S1).

Metagenomics
Per collection site 5 snow samples were taken. A snow sample set consists of three samples: layer A - fresh snow above the Saharan sand; layer B - snow containing Saharan sand; and layer C - snow below the Saharan sand layer (Fig.1). Depending on snow height on the two mountains Layer A was collected at 30 to 60cm, layer B at 70cm to 100cm and layer C at 120cm 180m under the snow surface. Additional six soil samples were taken at the Latemar collection site after snowmelt in a depth of approximately 10 to 20 cm under the surface. Snow was sampled wearing protective suits (coveralls), facemasks, and vinyl gloves. Surface snow and soil were removed using a clean sterilized shovel. Samples were collected in sterile plastic containers or plastic bags, transported on dry ice and stored at -80°C.
All samples were gently melted and opened inside a clean bench. Melted snow (approx. 30ml) was filtered (MO BIO Water filter; 0,22μm) and genomic DNA was extracted using the PowerWater® DNA Isolation Kit (MO BIO Laboratories Inc., Carlsbad, USA) according to the manufacturer's instructions. Soil samples were extracted using the FastDNA Spin Kit for Soil (MP Biomedicals, Santa Ana, USA) according to the manufacturer's instructions. DNA quality was assessed by gel electrophoresis and UV-Vis spectroscopy. Sequencing libraries of the variable V3-V5 region (F333 5'-TCCTACGGGAGGCAGCAG-3' and R934 5'- TGTGCGGGCCCCCGTCAATT-3') of the bacterial 16S rRNA gene and of the fungal ITS1 region (18SF 5'-GTAAAAGTCGTAACAAGGTTTC-3' and ITS1R 5'GTTCAAAGAYTCGATGATTCAC-3') were prepared using the FastStart High Fidelity PCR system (Roche Diagnostics, Mannheim, Germany) and the specific primers flanked with Roche Multiplex Identifier sequences. PCR was performed at an initial denaturation temperature of 95°C for 5min, followed by 30 (V3-V5) and 33 (ITS1) cycles of 95°C for 30sec (V3-V5) and 45sec (ITS1), annealing at 58°C for 30sec (V3-V5) and 45sec (ITS1), elongation at 72°C for 60sec (V3-V5) and 90sec (ITS1). A final elongation step at 72°C was run for 8min for the 16s rRNA gene V3-V5 region and for 10min for the ITS1 region. PCR products were analyzed by gel electrophoresis and purified using the AMPure XP beads kit (Beckman Coulter, Cassina De'Pecchi, Italy) following the manufacturer's instructions. After quantification by real time PCR using the Roche 454 titanium Library quantification kit (KAPA Biosystems, London, UK), samples were equimolarly pooled in a final amplicon library. Sequencing was performed on a GS FLX+ system using the XL+ chemistry following the manufacture's protocols.

Raw 454 files were demultiplexed using the Roche's sff file software, and are available at the European Nucleotide Archive (www.ebi.ac.uk/ena/) under the study accession number PRJEB11741. Sample accessions and metadata are available in Additional file 1: Table S3. Reads were pre-processed using the MICCA pipeline [49]. Forward and reverse primer trimming and quality filtering were performed using micca-preproc (parameters: -f TCCTACGGGAGGCAGCAG -r TGTGCGGGCCCCCGTCAATT -O 16 -l 400 -q 20 for the 16S rRNA gene and -f GTAAAAGTCGTAACAAGGTTTC -r GTTCAAAGAYTCGATGATTCAC -O

16 -l 200 -q 18 for ITS) truncating reads shorter than 400 nt for the 16S rRNA gene and 200 nt for ITS.

Operational taxonomic units (OTUs) were assigned by clustering the sequences with a threshold of 97% pair-wise identity using micca-otu-denovo (parameters for the 16S rRNAgene: -s 0.97 -c -d -o otus; for ITS: -s 0.97 -c -d -l 180 -o otus -t rdp --rdp-gene fungalits_unite --rdp-min-confidence 0.7), and their representative sequences were classified using the RDP [50] software version 2.8. As for the 16S rRNA gene, only samples with at least 3000 reads were considered. Samples were rarefied by subsampling to the 90% of depth of the least abundant sample.

Alpha (within-sample richness) and beta-diversity (between-sample dissimilarity) estimates were computed using the phyloseq R package [51]. Permutational MANOVA (PERMANOVA) statistical tests were performed using the R package vegan [52] (adonis function) with 9999 permutations.

To guarantee that OTUs present in layer B were not absent from layers A and C due to insufficient sampling we modelled the expected number of reads of each OTUs as a binomial process using the relative abundance in layer B as an estimate of the expected frequency, keeping only those for which the probability of zero count was <0.005.

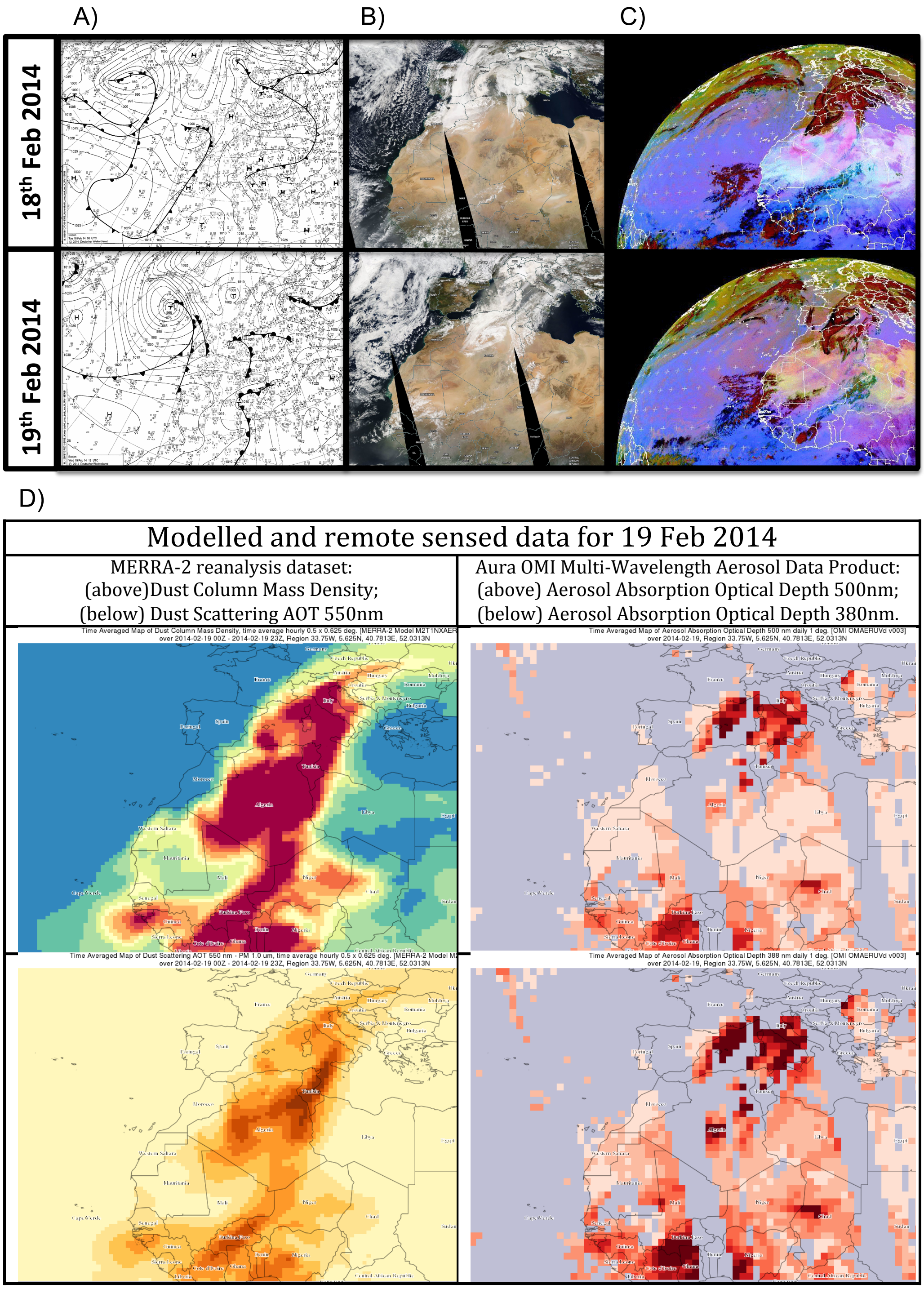

Additional file 1: Figure S1.

Synoptic description, satellite remote sensed images and numerical modeled fields of the massive dust plume that was deposed at ground in the Central Alpine region on 19th February 2014.
A) Synoptic chart with weather fronts at the surface: 18Feb2014 00 UTC and 19Feb2014 12 UTC.
B) Corrected Reflectance Terra/MODIS composite images (https://worldview.earthdata.nasa.gov)
C) Meteosat Second Generation satellite RGB-Dust images. The Dust RGB is composed from data from a combination of the SEVIRI IR8.7, IR10.8 and IR12.0 channels (From: http://sds-was.aemet.es)
D) Modelled and remote sensed data for 19 Feb 2014

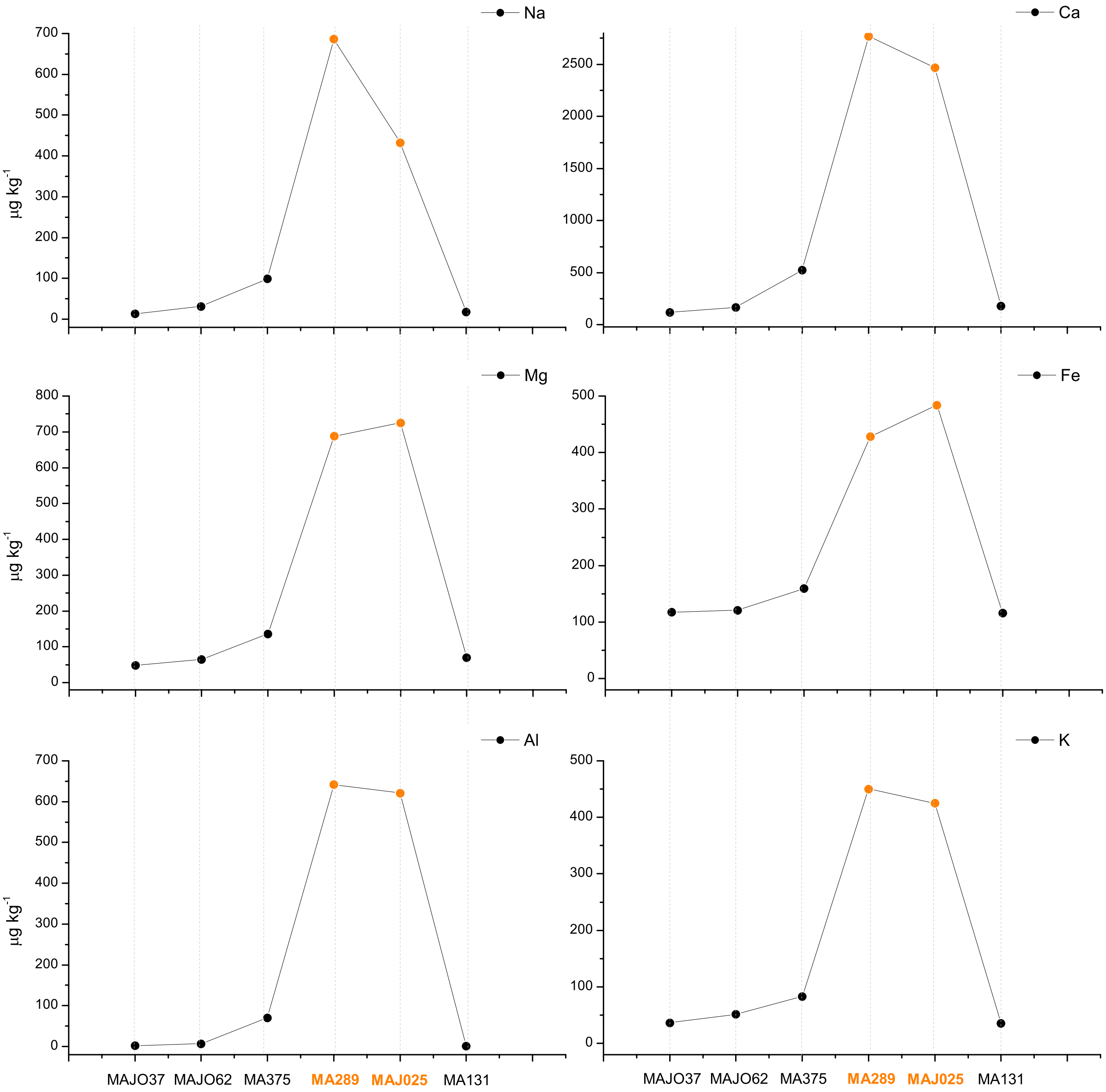

Additional file 1: Figure S2.

Crustal element concentrations in snow pit samples. Na, Ca, Mg, Fe, AL and K concentrations measured in snow pit samples from the Marmolada collection site. Samples containing Saharan dust particles, MA289 and MAJ025, are given in orange colour. The complete data set is reported in the Additional file 1: Table S1.

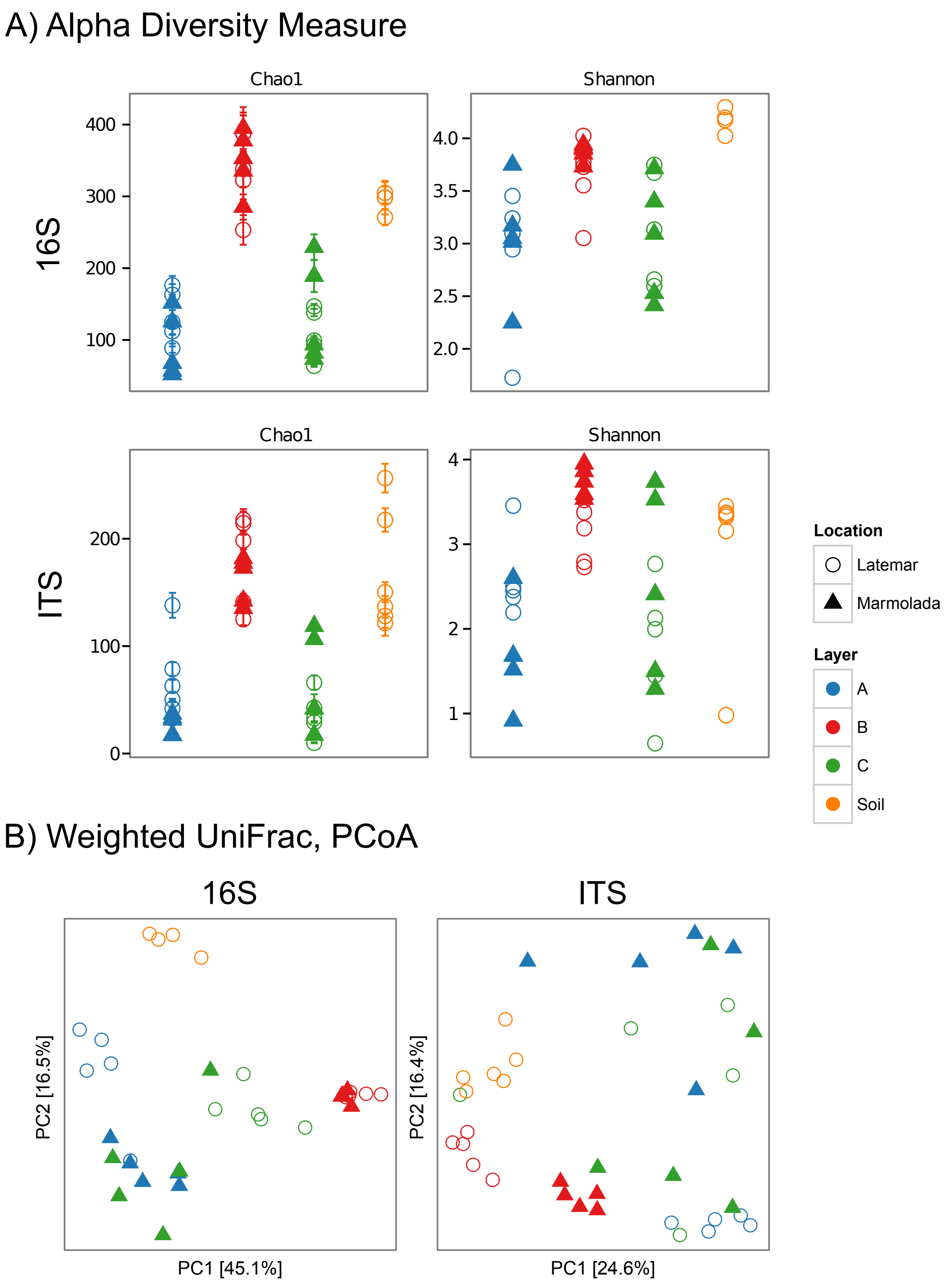

Additional file 1: Figure S3.

Community diversity between the three snow layers and soil samples. **(A)** The measures of the Chao1 estimator of species richness and the Shanon entropy are given for the bacterial 16S rRNA gene and the fungal ITS1 OTUs. Error bars indicate the Standard error SE. **(B)** PCoA of the between samples distances, for bacterial 16S rRNA gene and fungal ITS samples, were measured using weighted UniFrac distance (PERMANOVA, 9999 permutations; 16S rRNA gene: $P<0.0001$; ITS: $P<0.0001$). Symbol shapes indicate the two Dolomite mountain collection sides.

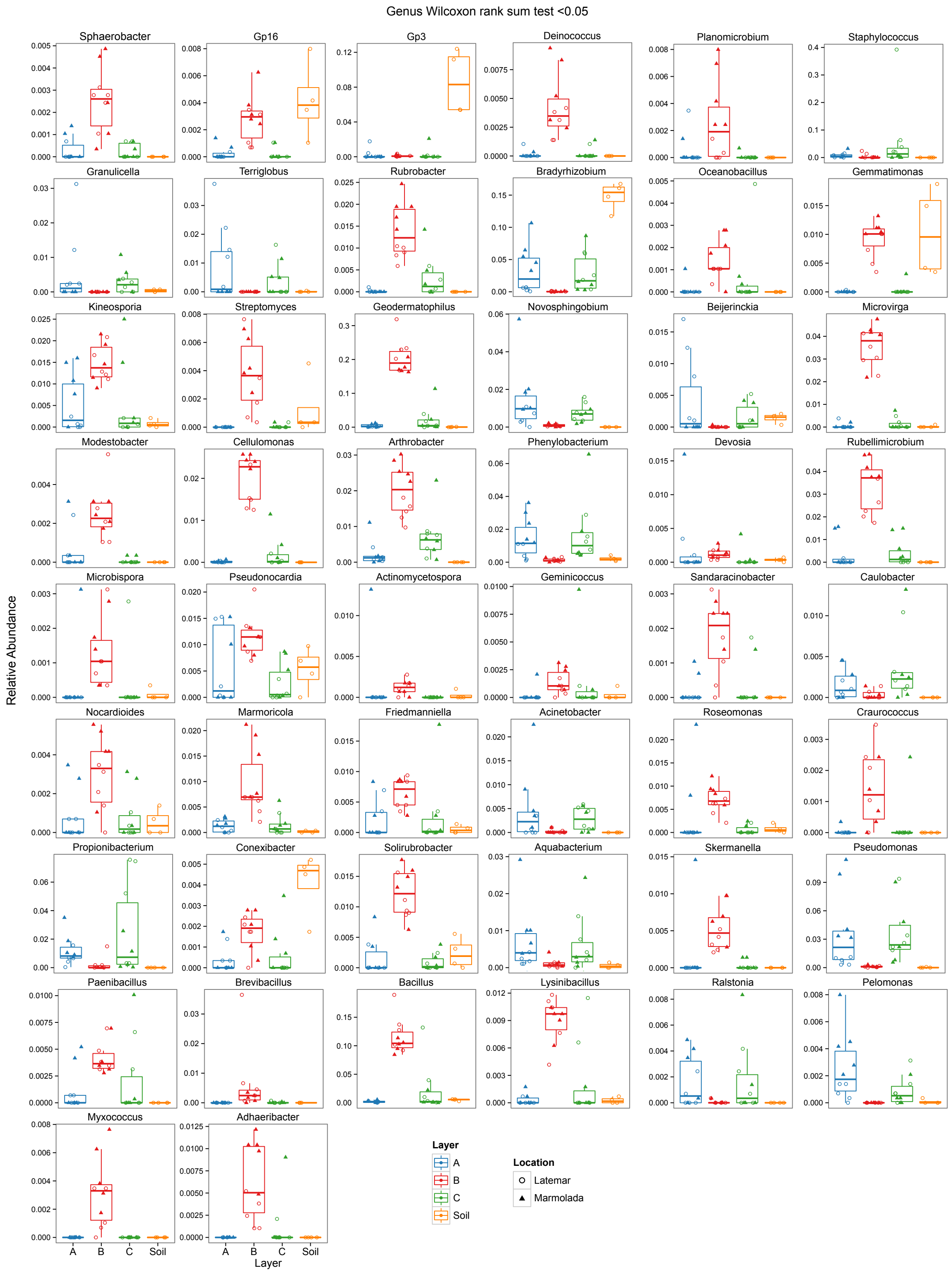

Additional file 1: Figure S4.

Significant enriched bacterial genera (Wilcoxon rank-sum test, P<0.05, FDR corrected) in snow layers. Comparison of the sand free snow layers A and C against the Saharan sand containing snow layer B. Principal bacterial genera described for Chad sand (Ref 22, main text) are enriched in layer B compared to layers A and C, namely *Geodermatophilus* ($P=7.58x10^{-5}$), *Arthrobacter* (P=0.00017), *Nocardiodes* (P=0.0027), *Rubrobacter* ($P=9.38x10^{-5}$), *Solirubrobacter* ($P=5.84x10^{-5}$) and *Bacillus* (P=0.00017). Genera taken in consideration are present >0,1% in at least 20% of all samples.

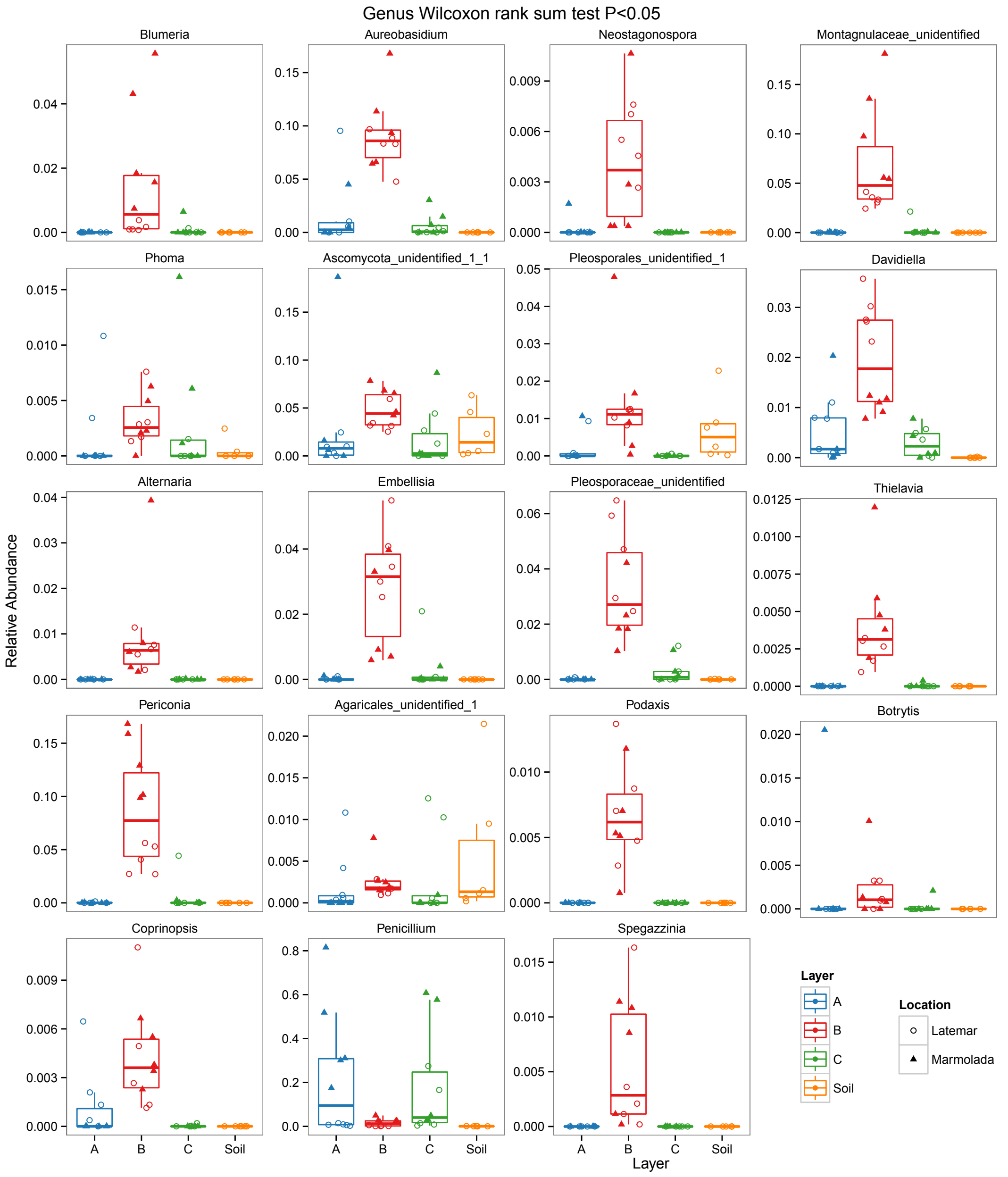

Additional file 1: Figure S5.

Significant enriched fungal genera (Wilcoxon rank-sum test, P<0.05, FDR corrected) in snow layers. Comparison of the sand free snow layers A and C against the Saharan sand containing snow layer B. Genera taken in consideration are present >1% in at least 20% of all samples.

Additional file 1: Table S1. Chemical composition of the snow samples collected in Marmolada. Concentrations are reported in µg/L. MA298 and MAJ025 (red shading) are the samples with visible presence of dust particles.

**Sample** (Depth in cm)	**MAJ037** (30)	**MAJ062** (60)	**MA375** (80)	**MA289** (85)	**MAJ025** (93)	**MA131** (120)
**Li**	0,0031	0,0054	0,0731	0,47	0,42	0,0028
**Na**	13,0	31	100	686	432	18
**Mg**	48	65	137	689	725	70
**Al**	2,0	6,8	70	642	620	1,6
**K**	36	51	83	450	425	36
**Ca**	120	169	524	2768	2468	181
**V**	0,024	0,022	0,25	1,5	1,5	0,013
**Cr**	0,41	0,41	0,52	0,86	1,04	0,40
**Mn**	0,13	0,36	12,5	31	27	0,33
**Fe**	118	121	160	428	484	116
**Co**	0,003	0,007	0,082	0,54	0,54	0,004
**Ni**	0,044	0,061	0,24	0,55	0,75	0,070
**Cu**	0,40	0,96	0,28	1,12	2,13	0,38
**Zn**	0,50	0,83	0,68	1,73	5,07	0,94
**Ga**	0,012	0,011	0,185	1,30	1,018	0,010
**As**	0,011	0,010	0,042	0,163	0,166	0,011
**Rb**	0,007	0,023	0,172	1,01	0,956	0,005
**Sr**	0,13	0,21	4,4	28	21	0,164
**Yb**	0,002	0,007	0,082	1,024	1,117	0,002
**Ag**	0,001	0,001	0,001	0,003	0,003	0,001
**Cd**	0,003	0,008	0,003	0,014	0,016	0,005
**In**	0,0002	0,0002	0,0003	0,0010	0,0011	0,0002
**Cs**	0,0006	0,0011	0,0073	0,0248	0,0406	0,0006
**Ba**	0,20	0,16	3,3	22	17	0,158
**La**	0,002	0,011	0,139	1,49	1,65	0,0009
**Ce**	0,0033	0,018	0,28	2,84	3,5	0,0015
**Pr**	0,0006	0,003	0,039	0,41	0,469	0,0004
**Nd**	0,0016	0,010	0,145	1,63	1,9	0,0012

**Sm**	0,0009	0,003	0,031	0,34	0,39	0,0009
**Eu**	0,0003	0,001	0,008	0,08	0,087	0,0003
**Gd**	0,0010	0,003	0,028	0,33	0,37	0,0005
**Tb**	0,0002	0,000	0,004	0,04	0,049	0,0002
**Dy**	0,0006	0,001	0,018	0,22	0,23	0,0005
**Ho**	0,0002	0,000	0,003	0,04	0,044	0,0002
**Er**	0,0006	0,001	0,009	0,10	0,109	0,0004
**Tm**	0,0001	0,000	0,001	0,01	0,014	0,0002
**Yb**	0,0004	0,001	0,007	0,07	0,077	0,0004
**Lu**	0,0001	0,000	0,001	0,01	0,011	0,0001
**Pb**	0,11	0,024	0,077	0,67	0,77	0,014
**Th**	0,001	0,002	0,022	0,028	0,025	0,001
**U**	0,000	0,001	0,008	0,072	0,072	0,001

Additional file 1: Table S3: Accuracy and precision of ICP-MS CRM measurements.

	**CRM certified concentrations** *µg/L*	**CRM found concentrations** *µg/L*	**Precision** *(%)*
Li	0,520 ± 0,136	0,45 ± 0,002	0,5
Be	0,387 ± 0,069	0,39 ± 0,004	1,1
Al	2,13 ± 0,754	2,50 ± 0,024	1,0
Ti	0,537 ± 0,187	0,47 ± 0,006	1,4
V	0,683 ± 0,090	0,59 ± 0,004	0,7
Cr	0,866 ± 0,165	0,83 ± 0,001	0,07
Mn	6,70 ± 0,660	6,42 ± 0,139	2,2
Fe	24,7 ± 4,12	23,59 ± 0,751	3,2
Co	0,246 ± 0,058	0,22 ± 0,002	0,9
Ni	0,908 ± 0,118	0,84 ± 0,010	1,2
Cu	7,03 ± 0,803	6,17 ± 0,021	0,1
Zn	8,47 ± 2,110	8,46 ± 0,054	0,2
As	1,14 ± 0,173	1,22 ± 0,027	2,23
Sr	1,82 ± 0,261	1,53 ± 0,017	1,1
Cd	0,524 ± 0,060	0,55 ± 0,002	0,4
Ba	0,870 ± 0,118	0,75 ± 0,006	0,8
Tl	0,377 ± 0,0599	0,33 ± 0,0001	0,04
Pb	0,346 ± 0,0695	0,28 ± 0,020	0,7

Additional file 1: Table S4: Comparison of significantly enriched bacterial genera in our study (Additional file 1: Fig. S4) with those described for different environments.

		Saharan dust							Other								
Weil et al.		[22]	[1]	[53]	[4]	[6]	[37]	[28]		[48]	[54]	[9]	[36]	[55]	[56]		
layer	genera	desert sand	airborne dust	airborne dust	airborne dust	snow + dust	historic samples	rain + dust	rain w/o dust	snow + Asian dust	perma-frost	glacier ice	glacier forefield	alpine forest soil	forest soil	grassland	desert
B	Myxococcus			■	■												
B	Adhaeribacter	■		■		■											
B	Rubrobacter	■		■	■						■						■
B	Streptomyces	■	■	■		■					■	■					
B	Modestobacter	■		■		■											
B	Cellulomonas	■	■			■						■					
B	Microvirga	■		■													
B	Arthrobacter	■	■	■		■		■			■		■	■		■	■
B	Paenibacillus	■	■	■			■			■	■		■	■			
B	Geodermatophilus	■		■		■			■								■
B	Rubellimicrobium	■		■		■		■	■								
B	Solirubrobacter	■		■		■		■									
B	Marmoricola	■		■													
B	Bacillus	■	■	■	■	■	■	■		■	■		■	■			
B	Microbispora										■						
B	Actinomycetospora																
B	Skermanella	■		■													
B	Lysinibacillus																
B	Sandaracinobacter																
B	Roseomonas	■															
B	Pseudonocardia	■		■		■						■					
B	Nocardioides	■	■	■		■											
B	Planomicrobium	■	■	■													
B	Kineosporia	■															
B	Friedmanniella			■													
B	Oceanobacillus	■						■									
B	Craurococcus	■															
B	Brevibacillus	■					■										
B	Deinococcus	■	■	■	■	■											
B	Sphaerobacter																
B	Devosia			■		■											
B	Geminicoccus			■													
B/Soil	Gemmatimonas			■	■				■		■		■				
B/Soil	Conexibacter	■				■											
B/Soil	Gp16			■	■						■						
Soil	GP3			■	■												
Soil	Bradyrhizobium	■		■		■									■	■	
A/C	Staphylococcus		■						■	■	■						
A/C	Granulicella														■	■	■
A/C	Terriglobus																
A/C	Novosphingobium		■	■		■			■								
A/C	Beijerinckia								■								
A/C	Phenylobacterium			■													
A/C	Caulobacter														■	■	■
A/C	Acinetobacter		■	■													
A/C	Propionibacterium		■														
A/C	Aquabacterium											■					
A/C	Pseudomonas	■	■	■		■		■	■	■	■	■		■	■	■	■
A/C	Ralstonia																
A/C	Pelomonas																

Comparison of bacterial genera significantly enriched in the analysed layers (Additional file 1: Fig. S4) with bacterial genera described in related studies, highlighting differences and similarities between genera associated to the Sahara (Saharan dust) and to other sources (Other). Layers are colour-coded according to Additional file 1: Fig. S4.

Additional file 1: References

53.
Katra I, Arotsker L, Krasnov H, Zaritsky A, Kushmaro A, Ben-Dov E. Richness and diversity in dust stormborne biomes at the southeast mediterranean. Sci Rep. 2014;4:5265.

54.
Wilhelm RC, Niederberger TD, Greer C, Whyte LG. Microbial diversity of active layer and permafrost in an acidic wetland from the Canadian High Arctic. Can J Microbiol. 2011;57:303–15

55.
Franca L, Sannino C, Turchetti B, Buzzini P, Margesin R. Seasonal and altitudinal changes of culturable bacterial and yeast diversity in Alpine forest soils. Extremophiles. 2016;20:855–73.

56.
Xu Z, Hansen MA, Hansen LH, Jacquiod S, Sørensen SJ. Bioinformatic Approaches Reveal Metagenomic Characterization of Soil Microbial Community. PLoS One. 2014;9:e93445.
